# Bovine upper alimentary squamous cell carcinoma associated with bracken fern poisoning: Clinical-pathological aspects and etiopathogenesis of 100 cases

**DOI:** 10.1371/journal.pone.0204656

**Published:** 2018-09-26

**Authors:** Tatiane Cargnin Faccin, Juliana Felipetto Cargnelutti, Fernando de Souza Rodrigues, Fernanda Rezer de Menezes, José Vitor Marcon Piazer, Stella Maris Pereira de Melo, Betina Fabis Lautert, Eduardo Furtado Flores, Glaucia Denise Kommers

**Affiliations:** 1 Departamento de Patologia, Universidade Federal de Santa Maria, Santa Maria, Rio Grande do Sul, Brazil; 2 Departamento de Medicina Veterinária Preventiva, Universidade Federal de Santa Maria, Santa Maria, Rio Grande do Sul, Brazil; 3 Instituto Federal Farroupilha, São Vicente do Sul, Rio Grande do Sul, Brazil; 4 Inspetoria Veterinária de Jaguari, Jaguari, Rio Grande do Sul, Brazil; Colorado State University, UNITED STATES

## Abstract

Upper digestive tract (UDT) cancer is rare in cattle, however in Southern Brazil, the UDT squamous cell carcinomas (SCCs) are relatively common and have been associated with bracken fern consumption and the presence of papillomas. Although a theory of pathogenesis considers bovine papillomavirus type 4 (BPV-4) as a cofactor in the development of these SCCs, some aspects of the etiopathogenesis of this disease need to be more investigated. In fact, detection of BPV-4 in UDT papillomas is scarce in other regions of the world and has not been performed in Brazil. Therefore, this study had two aims: 1) to analyze the epidemiological, clinical and pathological aspects of 100 natural cases of SCCs in the UDT of cattle grazing on bracken fern (*Pteridium arachnoideum*) highly contaminated areas, investigating the associations between these parameters; and 2) to investigate the presence of papillomavirus DNA by polymerase chain reaction (PCR) in the UDT papillomas (n = 47) from 30 cattle that also had UDT SCCs. There were statistically significant associations between clinical signs and tumor localization in the UDT; between histological grade of differentiation and tumor localization; and a trend towards significant association between histological grade of differentiation and presence of metastases. The average age of cattle with oropharyngeal SCCs was 7.39 years, with statistically significant difference comparing to cattle with esophageal SCCs (8.6 years). No statistical association was observed among other clinical-pathological parameters (growth pattern and primary site of the tumor) analyzed. No BPV DNA was detected in papillomas by PCR. Therefore, these results suggest the possibility that papillomas of the UDT are not necessarily associated with BPV infection.

## Introduction

Squamous cell carcinomas (SCCs) are the most common oral neoplasms of humans, cats, horses, and livestock species and are the second most common of dogs, behind only the melanocytic tumors [1–4]. Oral neoplasms are generally rare in livestock [3, 5]. An exception is seen in a few geographic areas, such as Southern Brazil, where oral, pharyngeal, esophageal, and ruminal SCCs may represent around 20% of all neoplasia of cattle [6]. In these regions, SCCs of the upper digestive tract (UDT) are associated with chronic bracken fern (*Pteridium* spp.) poisoning and presence of UDT papillomas [3, 5, 7–9].

Some studies show that UDT papillomas are caused by a productive bovine papillomavirus type 4 (BPV-4) infection [10, 11]. Although histological and electron microscopic data suggested a role of BPV-4 in the development of alimentary SCCs [7], and the disease has been experimentally reproduced [12], no BPV-4 DNA could be detected in almost all the SCCs tested by Southern blot hybridization [13] and studies using a more sensitive technique as polymerase chain reaction (PCR) have not been performed.

In Brazil, the relationship between these neoplasms and chronic ingestion of bracken fern is well established [6, 8, 9, 14], but there are no studies confirming the association of BPV-4 with this condition. Experimental reproduction of the disease is difficult to perform because cattle need to consume small amounts of the plant for several years to develop SCCs [15].

Therefore, this study had two different aims: 1) to analyze the epidemiological, clinical and pathological aspects of 100 natural cases of SCCs in the UDT of cattle grazing for years on bracken fern (*Pteridium arachnoideum*) highly contaminated areas in Southern Brazil, investigating the associations between these parameters; and 2) to investigate the presence of papillomavirus DNA in the UDT papillomas by PCR.

## Material and methods

Tumors from 100 cattle with SCCs in the UDT were collected between September 2003 and August 2014. Samples were obtained from necropsy examinations from our diagnostic laboratory. Age, sex, breed, clinical signs, tumor growth pattern and the frequency and localization of metastases were recorded. The primary site of the tumor was classified as base of the tongue, pharynx, epiglottis, proximal, middle and distal esophagus and rumen entrance. The localization of the tumors was classified as oropharyngeal (including base of the tongue, pharynx and epiglottis), esophageal (proximal, middle and distal esophagus) or ruminal (rumen entrance). When cattle had more than one SCC in the UDT, the largest tumor in centimeters was considered for this study [9]. The growth pattern (endophytic or exophytic) of the tumor was determined by visual examination with careful palpation on mucosa and cut surfaces [16].

Part of the papillomas (n = 47) from 30 cattle was collected and kept fresh or frozen for PCR. Another part of the papillomas and SCCs (n = 100) from 100 cattle were fixed in 10% buffered formalin, routinely processed for histopathology and stained with hematoxylin and eosin (HE). Microscopically, each SCC was graded as well, moderately, or poorly differentiated. Briefly, well differentiated SCCs were characterized mainly by islands of neoplastic keratinocytes with abundant eosinophilic cytoplasm, intercellular bridges, and concentric laminated deposits of keratin (keratin pearls). Nuclear pleomorphism and mitotic activity was minimal. Moderately differentiated SCCs were characterized by ribbons, cords or cellular aggregates of tumor cells, with less eosinophilic cytoplasm, nuclei showing greater pleomorphism and hyperchromatism and more numerous mitotic figures. Keratin pearls and intercellular bridges were less frequent. Poorly differentiated SCCs show little squamous differentiation. The cytoplasm appears amphophilic and the nuclei were pleomorphic, hyperchromatic and with marked mitotic activity. Neoplastic cells were deeply invasive, often appearing as single cells or small groups of cells surrounded by abundant desmoplastic reaction [14, 16].

### Ethics statement

In this study we did not perform any animal experiments. All the data were obtained from the archives of necropsy reports. All the samples (paraffinized and frozen) used in this work came from the archives of the diagnostic routine of the veterinary pathology laboratory.

### DNA extraction and PCR

Frozen (n = 42) and fresh (n = 5) fragments (50–100mg) of papillomas collected from 30 bovine oral or esophageal mucosa that also had UDT SCCs were submitted to total DNA extraction using the phenol and chloroform method. The samples were lysed and digested using 1x sodium dodecyl sulfate solution and 1mg/ml of proteinase K. The samples were incubated during 1–3h at 56°C. The organic and DNA phases were separated using phenol and chloroform solution and centrifugation (9,000 rpm, 30min). DNA precipitation was performed using ethanol and total DNA was eluted in 100μl of Tris-EDTA solution.

Total DNA was submitted to a PCR to amplify a 165 bp segment of the BPV-4 L1 gene. Initially, the sensitivity of the PCR was analyzed using the primers described by Borzacchiello et al. [11]. The positive control was a synthetic DNA containing the target sequence, that contains approx. 20 nucleotides plus flanking regions. The synthetic DNA was constructed using the complete sequence of BPV-4 deposited in Genbank (accession number X05817.1) as the model. This PCR allowed for the amplification of as low as 1.56x 10^6^ DNA copies. PCR reactions were performed in 25μl volume, using 2μl of template DNA, 12.5μM of each primer, 2.5mM of MgCl_2_, 10mM of dNTPs, 1x reaction buffer and 1 unit of Taq DNA polymerase (ThermoFischer Scientific). PCR conditions were: initial denaturation (95°C for 10 min), followed by 30 cycles of 95°C–60s; 45°C–60s for primer annealing and 72°C–60s for primer extension; and a final extension of 7 min at 72°C. Products were visualized in a 1.5% agarose gel, stained with Gel Red (Biotium, Inc., Fremont, CA) and visualized under UV light.

Panpapillomavirus PCR was also performed, using primers FAP59/64 and conditions described by Forslund et al. [17], and DNA of bovine papillomavirus type 1 extracted of a cutaneous wart was used as positive control. All reactions were performed using ultrapure water as negative control.

DNA of all extracted samples was initially submitted to endogenous control PCR to verify acid nucleic integrity. For this, partial sequence of housekeeping GAPDH gene (841bp) was amplified using primers forward (5’-3’: TGTTCCAGTATGATTCCAC) and reverse (3’-5’: TCCACCACCCTGTTGCTGTA).

### Statistical analysis

Statistical analysis and graphics were performed using R Core Team (2016) statistical software (R: a language and environment for statistical computing; R Foundation for Statistical Computing, Vienna, Austria). Chi-square or Fisher’s exact tests were used to examine the associations between clinicopathological parameters (clinical signs, growth pattern, tumor localization, primary site, metastases, and histological differentiation grade). Age and tumor localization were displayed as bar graphs. Average age by tumor localization was displayed as mean ± standard deviation. Age of bovines according to tumor localization was compared using the Student’s *t* test when data were normally distributed (determined with the Shapiro–Wilk test) or the Mann–Whitney U test when not normally distributed. A value of P <0.05 was considered statically significant.

## Results

The mean age of animals with UDT SCCs was 7.8 (±2.3) years, ranging from 3 to 13 years. Ninety-six were females and 4 were males. Eighty-nine were crossbreed (mixture of breeds), 6 Holstein, 4 Jersey and 1 Charolais.

The most common reported clinical signs were weight loss (n = 95), ruminal atony (n = 77), dysphagia (n = 62), coughing (n = 59), diarrhea (n = 53), regurgitation of ruminal contents through the mouth or nostrils (n = 36), bloating (n = 35), and salivation (n = 30). Less commonly signs were halitosis (n = 27), anorexia (n = 14), dyspnea (n = 8), neck extension (n = 6), weakness (n = 5) and kyphosis (n = 2). Sometimes, the presence of large amounts of undigested food in the feces was reported.

**Fig 1:**
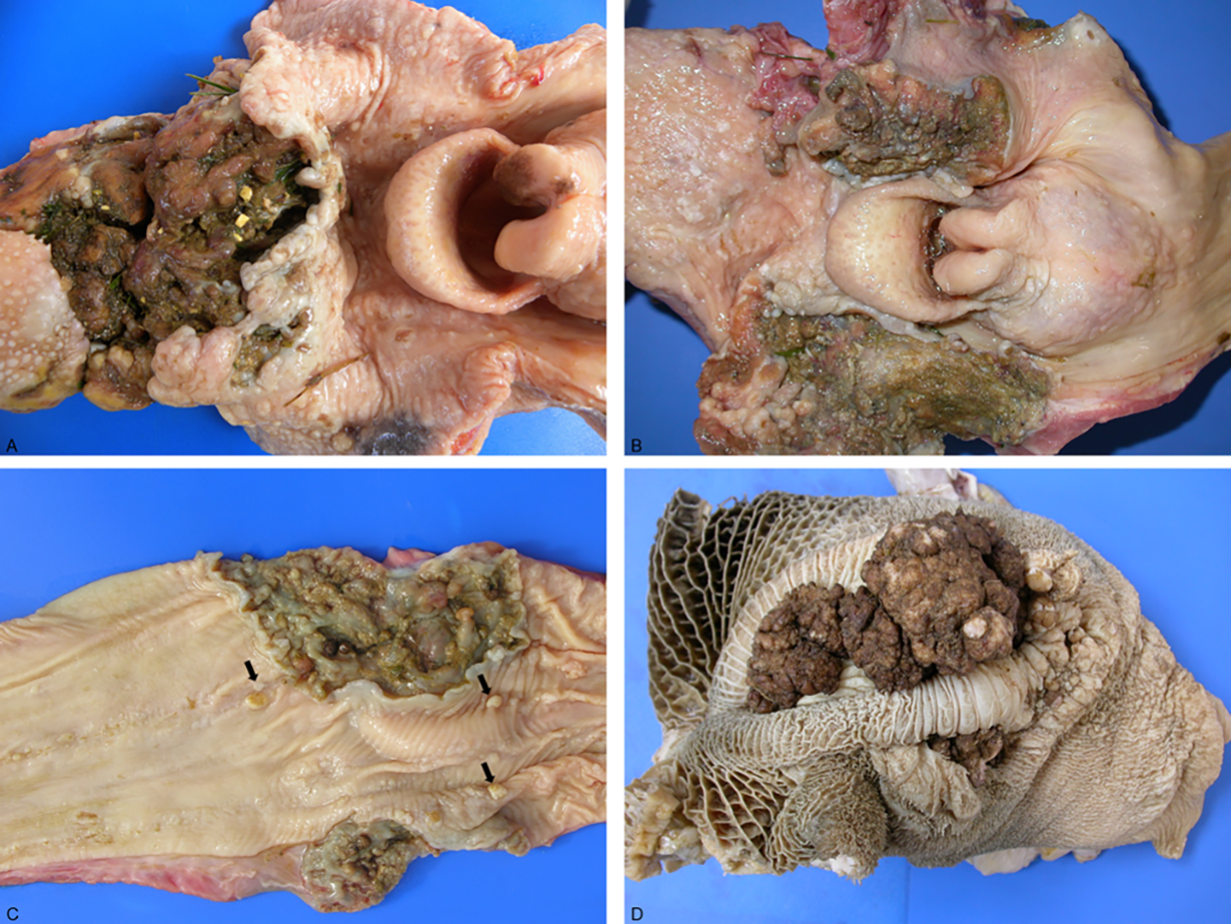
Squamous cell carcinoma of the upper digestive tract of cattle associated with chronic poisoning by bracken fern. Endophytic growth patterns are located in the base of the tongue (A), pharynx (B) and distal esophagus (C; including some small papillomas [arrows]) and exophytic pattern in the rumen (D).

The SCCs were more commonly observed in the oropharyngeal (n = 41; Fig 1A and 1B), followed by ruminal (n = 35) and esophageal (n = 24;) regions (Fig 1C and 1D) of the UDT. Of the 41 oropharyngeal tumors, 26 were located in the pharynx, 10 in base of the tongue and 5 in the epiglottis. Of the 24 esophageal tumors, 9 were in the proximal esophagus, 9 in the middle and 6 in the distal esophagus.

Eighty-one SCCs were of endophytic growth pattern and 19 were exophytic. The endophytic tumors were deeply infiltrating lesions, with relatively few surface manifestations (irregularities in the mucosa with small erosions or ulcers) or extensive ulcers (Fig 1A, 1B and 1C). The exophytic tumors were papillary or verrucous, irregular, ulcerated masses with a broad base or a relatively narrow pedicle (Fig 1D). Tumors ranged from 6 to 50 cm in extension, were multilobulated with retention of alimentary content and fetid odor. All SCCs were white-yellowish, firm masses, frequently with yellow spots on cut surface (corresponding to marked keratinization). Of all endophytic tumors, 13 esophageal tumors had a specific gross presentation as circumferential (annular) masses within the wall with pronounced wrinkling of the mucosa associated with retracted uneven areas and subsequent luminal stenosis (detailed data published elsewhere [18]).

In addition to the SCC large tumors, smaller tumors (<5cm in diameter) were observed, sometimes multifocally, along the UDT in 57 cattle. These small tumors had the same gross features of large tumors and were more frequent in the rumen entrance (n = 31) and esophagus (n = 27), followed by epiglottis (n = 17), base of tongue (n = 16), and pharynx (n = 12).

**Fig 2:**
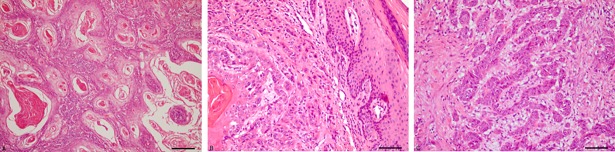
Histology of squamous cell carcinomas (SCCs) of the upper digestive tract of cattle associated with chronic poisoning by bracken fern. Well (A), moderately (B), and poorly (C) differentiated SCCs, HE.

**Fig 3:**
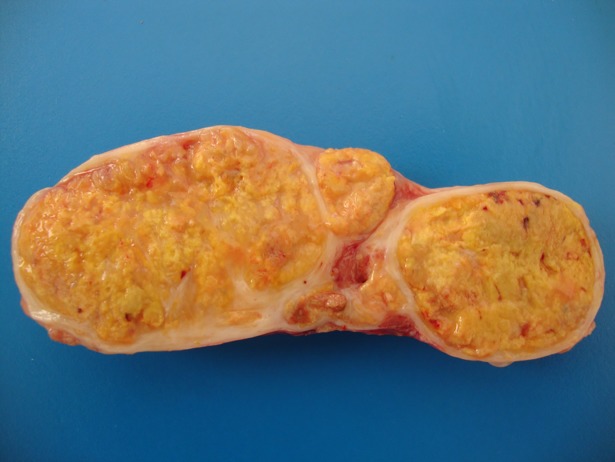
Regional lymph node metastasis of squamous cell carcinoma of the upper digestive tract of cattle associated with chronic poisoning by bracken fern. The yellow coloration is due to the abundant intratumoral keratinization.

The SCCs were graded as well (n = 62; Fig 2A), moderately (n = 21; Fig 2B) or poorly differentiated (n = 17; Fig 2C). Metastases were present in 52% of cases and these mostly involved regional lymph nodes (Fig 3; retropharyngeal [n = 27], gastric [n = 13], deep cervical [n = 10], mediastinal [n = 5], superficial cervical [n = 3], submandibular [n = 2], mesenteric [n = 2] and hepatic [n = 2]). Metastases to the liver (n = 7), lung (n = 5) and spleen (n = 4) were less common. Rarely (n≤2), metastases were observed in greater omentum, urinary bladder, intestine, kidney, trachea and diaphragm.

**Fig 4:**
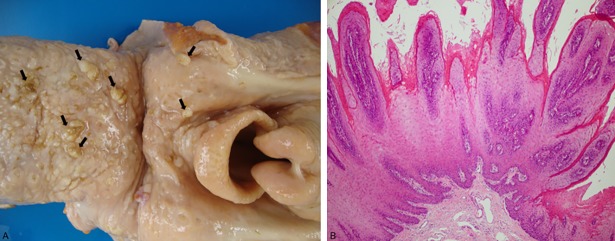
Papillomas associated with chronic poisoning by bracken fern on cattle. Multiple oropharyngeal papillomas (A; arrows) and developing papilloma (B), HE.

**Fig 5:**
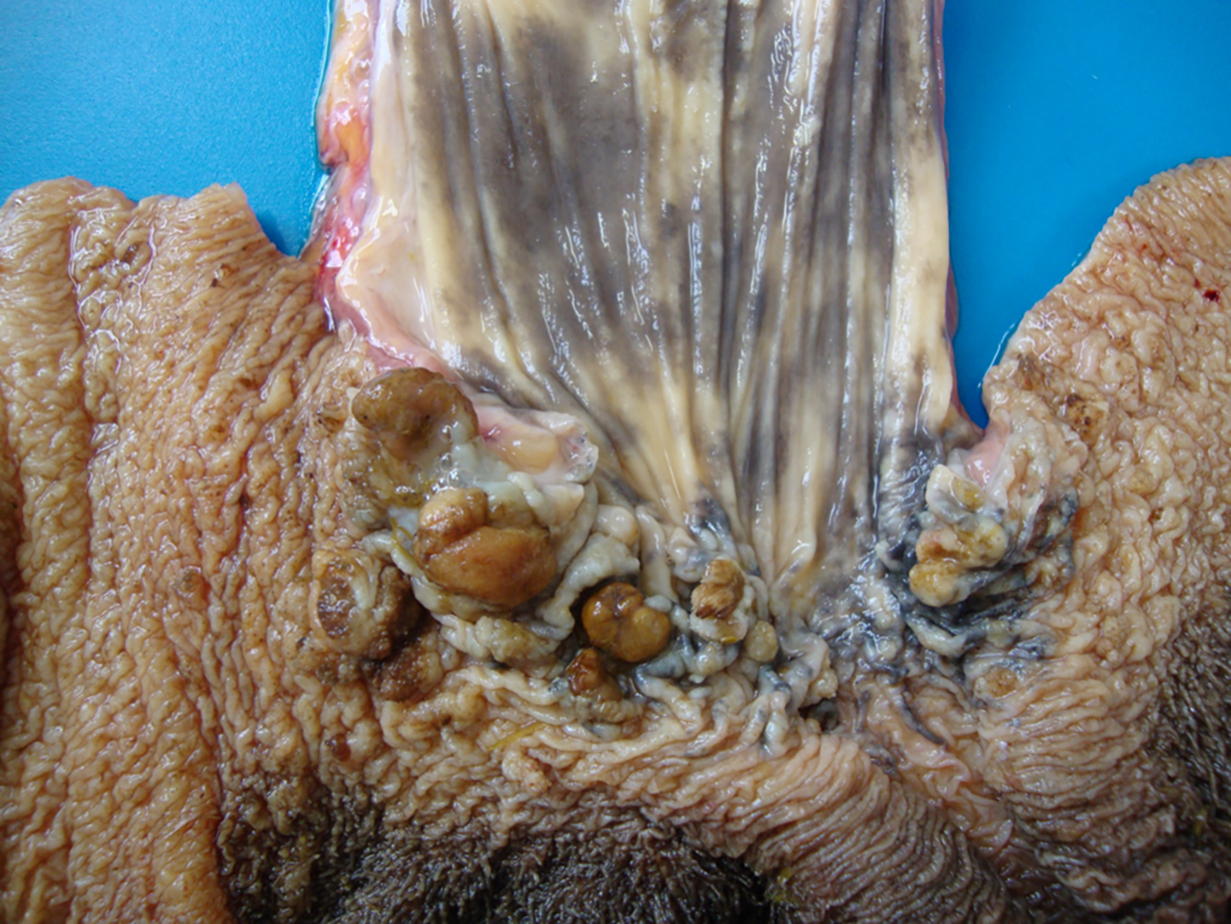
Ruminal transforming papillomas associated with chronic poisoning by bracken fern in a cow.

Throughout the UDT, from the soft palate to the rumen entrance, all 100 cattle had a few or multiple papillomas (Fig 4A), ranging from few millimeters in diameter to lesion that were up to 2 cm, pedunculate or sessile, with digitiform projections (cauliflower-like surface), and variable covering keratinization. Microscopically, papillomas were characterized by multiple digitiform projections covered by proliferated squamous epithelium with variable superficial keratinization, supported by a central fibroblastic stalk (Fig 4B). Intranuclear viral inclusion bodies were not observed. Three developing phases of papillomas were observed: growing (mild epithelial cupped hyperplasia, with initial formation of rete ridges in the lamina propria), developing (typical digitiform fronds with moderate to severe parakeratosis, anchored in a moderate collagenous stroma), and a few regressing papillomas (attenuated fronds with accentuation of rete ridges formation and moderate to severe infiltrate of lymphocytes). Sometimes, there were transforming papillomas, observed as an intermediate stage between a papilloma and a SCC. They were grossly characterized by attenuated digitiform projections (Fig 5) and histologically by papillomatous fronds showing areas of irregular growth and transformed keratinocytes.

Grossly, adjacent to the SCCs, the mucosa was often irregular, with focal patches of increased prickle layer or nodules with markedly irregular surfaces, small erosions or ulcers. Histologically, these squamous intraepithelial lesions were characterized by squamous hyperplasia, mild, moderate or severe dysplasia and carcinomas in situ. Moderate to severe dysplasia and carcinoma in situ were frequently accompanied by moderate lymphoplasmacytic inflammatory infiltrate.

PCR failed to amplify a segment of the BPV genome from the (n = 47 papillomas of 30 cattle) extracted samples. The BPV-4 PCR was able to detect at least 1.56 x 10^6^ DNA copies. The same was observed in panpapillomavirus PCR (FAP59/64) using bovine papillomavirus type 1 as positive control. Furthermore, the total DNA integrity of all analyzed samples was preserved, and it was confirmed by a PCR using the GAPDH *housekeeping* gene as target.

A significant (p<0.001) association between clinical signs and tumor localization was observed (Table 1). Coughing is the clinical sign associated with oropharyngeal tumors, regurgitation is related to esophageal neoplasms and bloating with ruminal SCCs.

**Table 1:** Association between clinical signs and SCCs localization in the upper digestive tract in cattle grazing on bracken fern areas.

	Tumor localization		
	Oropharyngeal (n = 41)	Esophageal (n = 24)	Ruminal (n = 35)
Clinical signs*			
**Weight loss: n = 95**	41 (43.16%)	22 (23.16%)	32 (33.68%)
**Ruminal atony: n = 77**	32 (41.56%)	17 (22.08%)	28 (36.36%)
**Dysphagia: n = 62**	26 (41.93%)	16 (25.81%)	20 (32.26%)
**Coughing: n = 59**	32 (54.24%)	12 (20.34%)	15 (25.42%)
**Diarrhea: n = 53**	16 (30.19%)	14 (26.41%)	23 (43.40%)
**Regurgitation: n = 36**	8 (22.22%)	17 (47.22%)	11 (30.56%)
**Bloating: n = 35**	3 (8.57%)	10 (28.57%)	22 (62.86%)
**Salivation: n = 30**	14 (46.66%)	8 (26.67%)	8 (26.67%)

*Significant (p<0.001) association by Chi-square or Fisher’s exact tests between tumor localization and clinical signs.

The association between histological differentiation grade and tumor localization was statistically significant (p = 0.007; Table 2). The well differentiated tumors were more oropharyngeal, and the poorly differentiated tumors were more often observed in esophageal and ruminal regions. There was a trend towards significance (p = 0.063) association between the variables histological differentiation grade and presence of metastasis (Table 2). Poorly differentiated SCCs had the highest rates of metastases (76.47%; 13/17) when compared to moderately (52.38%) and well (45.16%) differentiated SCCs.

**Table 2:** Association between histological differentiation grade and tumor localization or presence of metastasis of SCCs of the upper digestive tract in cattle grazing in bracken fern areas.

	Histological differentiation grade		
	WD (n = 62)	MD (n = 21)	PD (n = 17)
**Tumor localization**^a^			
**Oropharyngeal: n = 41**	32 (78.05%)	8 (19.51%)	1 (2.44%)
**Esophageal: n = 24**	11 (45.83%)	6 (25%)	7 (29.17%)
**Ruminal: n = 35**	19 (54.29%)	7 (20%)	9 (25.71%)
**Metastases**^b^			
**Presence: n = 52**	28 (53.85%)	11 (21.15%)	13 (25%)
**Absence: n = 48**	34 (70.84%)	10 (20.83%)	4 (8.33%)

WD, well-differentiated; MD, moderately differentiated; PD, poorly differentiated

^a^Significant (p = 0.007) association by Chi-square or Fisher’s exact tests between histological differentiation grade and tumor localization

^b^Trend towards significance (p = 0.063) association by Chi-square or Fisher’s exact tests between the variables histological differentiation grade and presence of metastasis.

Oropharyngeal tumors metastasized in 62.5% of cases, ruminal in 48.57% and esophageal tumors in 41.67% of cases. Despite this, tumor localization (oropharyngeal, esophageal and ruminal) was not significantly associated with the presence of metastasis (p = 0.284).

Considering the primary site, epiglottis tumors metastasized in 80% of cases; n = 4/5, followed by those located in the distal esophagus (67%; n = 4/6), pharynx (61.5%; n = 16/26), tongue (50%; n = 5/10), rumen entrance (48.5%; n = 17/35) and cranial and middle esophagus (33%; n = 3/9). However, no significant association between tumor primary site and presence of metastasis (p = 0.486) was observed.

**Fig 6:**
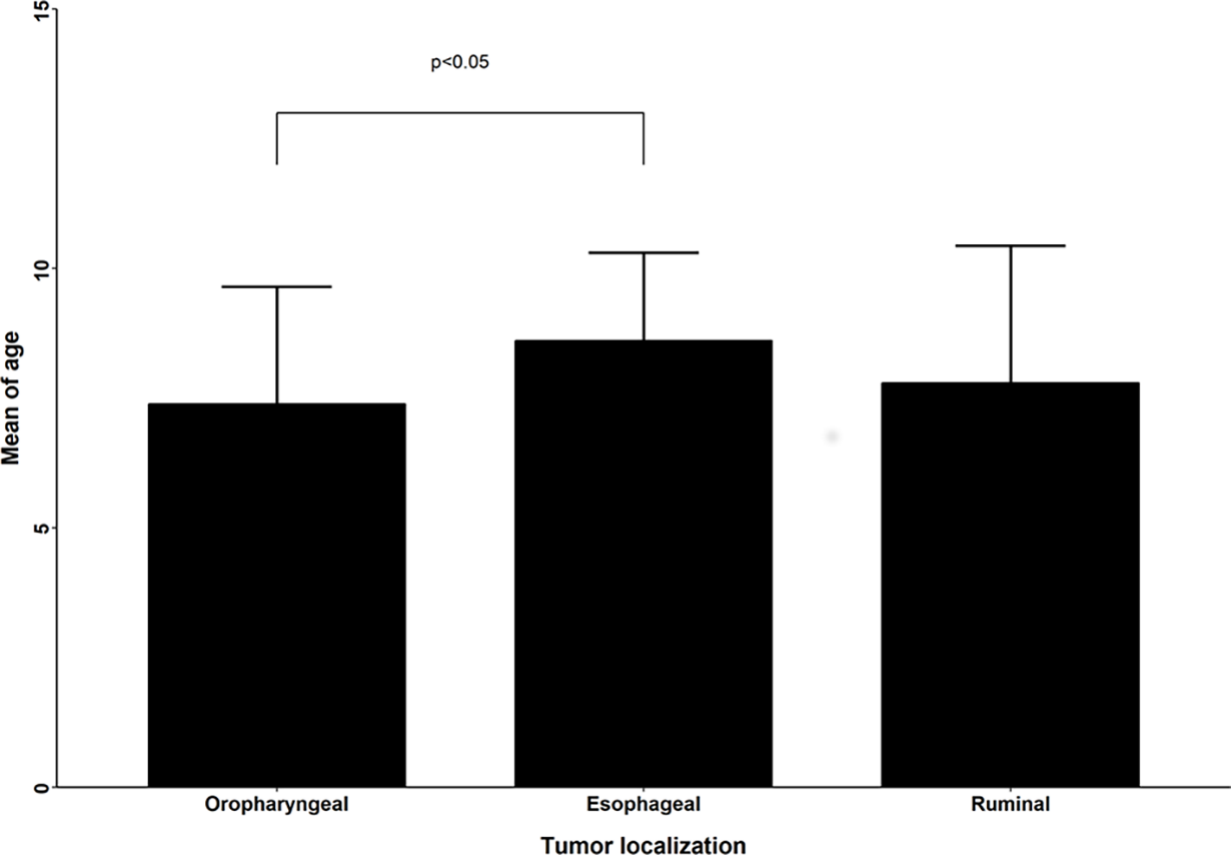
Squamous cell carcinomas (SCCs) of the upper digestive tract (UDT) in cattle associated with chronic poisoning by bracken fern. The average age of cattle according to tumor localization in the UDT. Cattle with oropharyngeal SCCs (7.39 years old) were younger than those with esophageal SCCs (8.6 years, *p = 0.018). Although cattle with oropharyngeal SCCs were, on average, younger than those with ruminal SCCs (7.79 years), the difference was not statistically significant.

The average age of cattle with oropharyngeal SCCs was 7.39 years old, with statistically significant difference (p = 0.018; *t* test) than cattle with esophageal SCCs (8.6 years). No significant difference was found among the mean age of cattle with esophageal and ruminal tumors (7.79 years) or oropharyngeal and ruminal tumors (Fig 6).

No statistical association was observed among other clinical-pathological parameters (growth pattern and primary site of the tumor) analyzed.

## Discussion

One hundred cases of SCCs of the UDT of cattle grazing on bracken fern in Southern Brazil were studied. Studies analyzing other aspects of the disease, with 30 [8] and 40 cases [14, 19], out of the 100 studied here, were already published.

Cattle with oropharyngeal SCCs were significantly younger than cattle with esophageal tumors; however, the reason for this was not determined. Cranial neoplasms may progress more rapidly than tumors of other sites in the UDT, or the clinical signs are more rapidly detectable in cranial SCCs [20].

Although cows are overrepresented in the present study, this does not represent a predisposition for females to develop these tumors. It can be explained by the fact that this category represents the largest bovine population on farms of that geographic area and also because cows have a longer life than the other categories of cattle, being more likely to develop the lesions of chronic poisoning by bracken fern [8]. According to the literature, SCCs associated with chronic bracken fern poisoning takes several years to develop [12, 15]. This is the possible reason the disease was not observed in cattle younger than 3-years-old in this study (mean age of 7.8), considering that many cattle were exposed to and probably consumed the plant since they were born in the affected farms.

Cachexia, ruminal atony and dysphagia were the most common clinical signs in cattle grazing on bracken fern areas, similar to that already observed by other authors [5, 9]. There was a significant association between clinical signs and tumor localization. We observed that coughing is the clinical sign associated with oropharyngeal tumors, regurgitation is related to esophageal tumors and bloating with ruminal neoplasms. Dysphagia was a common clinical sign, regardless of tumor localization. Oropharyngeal SCCs should be highly considered in the differential diagnosis of cattle with coughing grazing on bracken fern infested areas. Esophagus and stomach deserve particular attention during the examination of animals with regurgitation. The bloating observed in ruminal tumors was the result of a physical obstruction, mainly of the esophageal groove, in eructation of gas produced by normal rumen fermentation (chronic or recurrent secondary bloating) [5].

Histological differentiation grade was the main factor predicting the prognosis of SCCs in the UDT of cattle. The poorly differentiated tumors were more associated with the presence of metastasis. Although the prognostic value of histological classification of oral SCCs remains a controversial topic in human medicine and there have been a few studies to determine whether the histological grade also is prognostic in other domestic animals, this should be further evaluated because it is possible that the subtype of the SCCs and histological grade may be useful to predict tumor behavior, especially in cases in which complete clinical staging is not possible [3, 21]. There was also a significant association between histological differentiation grade and tumor localization in the UDT. The well-differentiated tumors were most oropharyngeal, and the poorly differentiated tumors were most in the esophageal and ruminal regions, however, the reason for this was not determined.

The site of the primary tumor is predictive of nodal metastasis and prognosis in human SCCs [22]. Human SCCs also are well recognized to metastasize more quickly from the tongue than from other areas of the oral cavity, probably due to the rich vascular and lymphatic concentration within the tongue and the contraction of tongue muscles promoting the dissemination of neoplastic cells [3, 22]. Comparing with neoplasms that develop at the base of the tongue, rostral SCCs in people have a more favorable prognosis due to more rapid detection, a greater chance of surgical cure, and, because there are fewer lymphatics in the rostral tongue, resulting in less frequent metastases. Similarly, less well-vascularized areas, such as the glottic larynx, are associated with a lower rate of metastasis [3, 22]. In dogs, although neoplasia of the tongue is rare, representing 3–6% of canine oral neoplasia, these neoplasms may have increased metastatic potential, with up to 40% of lingual SCCs reported to metastasize to regional lymph nodes compared to metastasis rates 15% for other oral SCCs [3]. In the present study, tumors on the rostral tongue were not observed. All tumors occurred at the base of the tongue. Although the rates of metastases were different between oropharyngeal, esophageal and ruminal tumors, and also between the different sites of the oral cavity, there was no association between the presence of metastasis and tumor localization in the UDT or tumor primary site in our study.

SCCs of oral cavity occurred more frequently in the pharynx (63.41%; 26/41), followed by the base of the tongue (24.39%; 10/41) and epiglottis (12.2%; 5/41). In humans, SCCs occur mainly in the oral cavity (67%) followed by pharyngeal region (30%) [1], it is suggested that higher exposure to tobacco smoking and alcohol drinking are required to induce oropharyngeal than oral cancer [23]. It is important to point out that the main toxic principle of bracken fern, a norsesquiterpene ptaquiloside, unfolds into a reactive dienone (carcinogenic and mutagenic) in places of alkaline pH, as under influence of saliva [24, 25].

Metastases were present at a much higher frequency (52% of cases) in our study when compared to oral SCCs in dogs and cats (around 15% of nodal metastasis) [3, 20]. Regional lymph node metastasis is most often found with poorly differentiated SCCs or neoplasms that have been present for a long time before they are diagnosed or excised [16]. Poorly differentiated SCCs had the highest rates of metastases in this study. Additionally, the SCCs in cattle are usually diagnosed late, when the animal shows evident clinical signs due to large or critically located (i.e. in the rumen entrance) neoplastic masses. Therefore, these two factors may have contributed to the high frequency of metastases in the present study.

Endophytic SCCs were much more frequent (81% of cases) than exophytic tumors, in accordance with the local invasive nature of this tumor, but no statistical association was observed between the growth pattern and clinical or pathological factors. Of all endophytic tumors, 13 had a specific gross presentation as annular stenotic esophageal SCCs, which were detailed elsewhere [18]. In humans, head and neck endophytic SCCs have worst prognosis than exophytic tumors [26–29]. Nevertheless, other studies have established that thickness of the tumor is the most reliable predictive factor that had significant value for subclinical nodal metastasis, local recurrence, and survival in patients with oral cancer [30–32]. The tumor thickness is measured from the deepest tumor invasion to the presumed original surface level, that is, ignoring exophytic growth or assessing the original surface level in ulcerated tumors [2, 30]. Additionally, the higher incidence of subclinical nodal metastasis and poor prognosis found in endophytic and expansive tumors in some studies can be probably caused by their association with thicker tumors in humans [32]. More studies are needed to determine if tumor thickness also influences the prognosis of SCCs in cattle.

The presence of BPV DNA was investigated in papillomas by PCR with primers specific for BPV-4 and with panpapillomavirus degenerate primer. Despite the relatively low sensitivity of the PCR used in this study, high copy numbers of BPV-4 DNA sequences have been regularly detected in both naturally occurring or experimentally induced papillomas, as observed by Campo et al. [13] who detected BPV-4 DNA in 61 out of 67 papillomas in which the copy number could be as high as 1 x 10^5^ genomes per cell, indicating the presence of high viral load in infected tissues.

The absence of DNA detection of BPV-4 by PCR and the absence of intranuclear inclusion bodies in papillomas suggest that papillomas of the UDT in cattle may not be necessarily associated with papillomavirus infection in Southern Brazil.

Additionally, the presence of squamous intraepithelial lesions found throughout the UDT epithelium that do not appear to pass through a papillomatous stage may demonstrate direct action of bracken fern carcinogens in the epithelium without the need for viral involvement [33]. In Brazil, both SCCs and papillomatosis of the UDT occur only in areas with presence of bracken fern [15].

Oral papillomatosis in cattle is rare and it is observed in specific cases of immunosuppression [34]. According to the papilloma-carcinoma theory proposed by Campo et al. [12], persistence of alimentary papillomatosis, with progression to SCCs, is induced by immunosuppression due to lymphopenia caused by *Pteridium* spp. toxicity. In a study of spontaneous cases of bracken fern-related SCCs of the UDT in Southern Brazil, lymphopenia was observed only in three out of 40 cases [19].

The absence of papillomavirus DNA in the papillomas, the absence of lymphopenia in most cows and the presence of squamous intraepithelial lesions in the UDT, keep open the possibility that papillomas of the UDT may not be necessarily associated with papillomavirus in cattle from our region and that papillomas and SCCs could be both independently induced by bracken fern. This possibility was also considered in a broad bovine and human comparative papillomavirus review [35]. Consequently, BPV-4 infection apparently is not a cofactor involved in carcinogenesis of alimentary SCCs in cattle from Southern Brazil. All these findings point out to the need for further investigations of the etiopathogenesis of this disease using other molecular and antigenic tests, such as next generation sequencing and BPV-4 specific monoclonal antibodies.

## Supporting information

S1 File In the absence of a commercially available specific primary antibody for BPV-4, a monoclonal antibody cocktail against human papillomavirus was used by immunohistochemistry and nuclear immunoreactivity for papillomavirus was not observed in any (n = 93 papillomas of 38 cattle) of the UDT papillomas (Figure in S1A and S1B Fig). (PDF)

S1 Fig Immunohistochemistry of bovine papillomas using a human papillomavirus cocktail broad spectrum (HPV-1, 6, 11, 16, 18 and 31), clone BPV-1/1H8 + CAMVIR-1 as primary antibody. Nuclear immunoreactivity within the superficial keratinocytes in a bovine cutaneous papilloma used as positive control. Inset shows an immunolabelled keratinocyte (A). Absence of immunoreactivity in a bovine upper digestive tract papilloma. Inset shows absence of immunostaining in epithelial cells (B).
(TIF)

## References

[1] Canto MT, Devesa SS. Oral cavity and pharynx cancer incidence rates in the United States, 1975–1998. Oral Oncol. 2002;38(6):610–7. Epub 2002/08/09. . 12167440 10.1016/s1368-8375(01)00109-9

[2] Johnson N, Franceschi S, Ferlay J, Ramadas K, Schmid S, MacDonald DG, et al Squamous cell carcinoma of the oral cavity and oropharynx In: Barnes L, Eveson J, Reichart P, Sidransky D, editors. Pathology and Genetics of Head and Neck Tumours. World Health Organization Classification of Tumours. Lyon: IARCPress; 2005 pp. 168–75.

[3] Munday JS, Löhr CV, Kiupel M. Tumours of the alimentary tract. In: Meuten DJ, editor. Tumours in Domestic Animals. 5 ed Ames: John Wiley & Sons Inc.; 2017 pp. 499–601.

[4] Stebbins KE, Morse CC, Goldschmidt MH. Feline oral neoplasia: a ten-year survey. Vet Pathol. 1989;26(2):121–128. Epub 1989/03/01. 10.1177/030098588902600204 . 2711569

[5] Uzal FA, Plattner BL, Hostetter JM. Alimentary system In: Maxie MG, editor. Jubb, Kennedy, and Palmer’s Pathology of Domestic Animals. vol 2 6 ed St. Louis: Elsevier; 2016 pp. 1–257.

[6] Lucena RB, Rissi DR, Kommers GD, Pierezan F, Oliveira-Filho JC, Macedo JT, et al A retrospective study of 586 tumours in Brazilian cattle. J Comp Pathol. 2011;145(1):20–24. Epub 2011/01/21. 10.1016/j.jcpa.2010.11.002 . 21247583

[7] Jarrett WF, McNeil PE, Grimshaw WT, Selman IE, McIntyre WI. High incidence area of cattle cancer with a possible interaction between an environmental carcinogen and a papilloma virus. Nature. 1978;274:215–217. Epub 1978/07/20. . 210386 10.1038/274215a0

[8] Souto MAM, Kommers GD, Barros CSL, Piazer JVM, Rech RR, Riet-Correas F, et al Neoplasms of the upper digestive tract of cattle associated with spontaneous ingestion of bracken fern (*Pteridium aquilinum*). Pesq Vet Brasil. 2006;26(2):112–22. 10.1590/S0100-736x2006000200009. WOS:000238575700009.

[9] Tokarnia CH, Döbereiner J, Canella CFC. Ocorrência de hematúria enzoótica e de carcinomas epidermóides no trato digestivo superior em bovinos no Brasil. II. Estudos complementares. Pesqui Agropec Bras. 1969;4:209–24.

[10] Campo MS, Moar MH, Jarrett WF, Laird HM. A new papillomavirus associated with alimentary cancer in cattle. Nature. 1980;286:180–182. 10.1038/286180a0 . 6250043

[11] Borzacchiello G, Ambrosio V, Roperto S, Poggiali F, Tsirimonakis E, Venuti A, et al Bovine papillomavirus type 4 in oesophageal papillomas of cattle from the south of Italy. J Comp Pathol. 2003;128(2–3):203–206. Epub 2003/03/14. . 12634101 10.1053/jcpa.2002.0626

[12] Campo MS, O'Neil BW, Barron RJ, Jarrett WF. Experimental reproduction of the papilloma-carcinoma complex of the alimentary canal in cattle. Carcinogenesis. 1994;15(8):1597–601. Epub 1994/08/01. . 8055638 10.1093/carcin/15.8.1597

[13] Campo MS, Moar MH, Sartirana ML, Kennedy IM, Jarrett WF. The presence of bovine papillomavirus type 4 DNA is not required for the progression to, or the maintenance of, the malignant state in cancers of the alimentary canal in cattle. EMBO J. 1985;4(7):1819–25. Epub 1985/07/01. ; PubMed Central PMCID: PMCPMC554423. 2992946 10.1002/j.1460-2075.1985.tb03856.x PMC554423

[14] Masuda EK, Kommers GD, Martins TB, Barros CS, Piazer JV. Morphological factors as indicators of malignancy of squamous cell carcinomas in cattle exposed naturally to bracken fern (*Pteridium aquilinum*). J Comp Pathol. 2011;144(1):48–54. Epub 2010/06/15. 10.1016/j.jcpa.2010.04.009 . 20542519

[15] Tokarnia CH, Brito MF, Barbosa JD, Peixoto PV, Döbereiner J. Plantas Tóxicas do Brasil para Animais de Produção. 2 ed Rio de Janeiro: Helianthus; 2012.

[16] Goldschmidt MH, Goldschmidt KH. Epithelial and melanocytic tumors of the skin. In: Meuten DJ, editor. Tumours in Domestic Animals 5 ed Ames: John Wiley & Sons Inc.; 2017 pp. 88–141.

[17] Forslund O, Antonsson A, Nordin P, Stenquist B, Hansson BG. A broad range of human papillomavirus types detected with a general PCR method suitable for analysis of cutaneous tumours and normal skin. J Gen Virol. 1999;80 (Pt 9):2437–2443. Epub 1999/09/29. 10.1099/0022-1317-80-9-2437 . 10501499

[18] Faccin TC, Masuda EK, Piazer JVM, Melo SMP, Kommers GD. Annular stenotic oesophageal squamous cell carcinoma in cattle exposed naturally to bracken fern (*Pteridium arachnoideum*). J Comp Pathol. 2017;157(2–3):174–180. Epub 2017/09/25. 10.1016/j.jcpa.2017.07.008 . 28942300

[19] Masuda EK, Kommers GD, Rosa FB, Barros CSL, Fighera RA, Piazer JVM. Relationship between lymphopenia and the persistence of alimentary papillomatosis in cattle chronically and spontaneously poisoned by bracken fern (*Pteridium aquilinum*). Pesq Vet Brasil. 2011;31(5):383–388. 10.1590/S0100-736x2011000500004. WOS:000293007000004.

[20] Martin CK, Tannehill-Gregg SH, Wolfe TD, Rosol TJ. Bone-invasive oral squamous cell carcinoma in cats: pathology and expression of parathyroid hormone-related protein. Vet Pathol. 2011;48(1):302–312. Epub 2010/10/14. 10.1177/0300985810384414 ; PubMed Central PMCID: PMCPMC4519039. 20940448 PMC4519039

[21] Nemec A, Murphy B, Kass PH, Verstraete FJ. Histological subtypes of oral non-tonsillar squamous cell carcinoma in dogs. J Comp Pathol. 2012;147(2–3):111–120. Epub 2012/02/04. 10.1016/j.jcpa.2011.11.198 . 22300705

[22] Genden EM, Ferlito A, Bradley PJ, Rinaldo A, Scully C. Neck disease and distant metastases. Oral Oncol. 2003;39(3):207–212. Epub 2003/03/06. . 12618192 10.1016/s1368-8375(02)00049-0

[23] Franceschi S, Bidoli E, Herrero R, Munoz N. Comparison of cancers of the oral cavity and pharynx worldwide: etiological clues. Oral Oncol. 2000;36(1):106–115. Epub 2000/07/13. . 10889929 10.1016/s1368-8375(99)00070-6

[24] Fenwick GR. Bracken (*Pteridium aquilinum*)-toxic effects and toxic constituents. J Sci Food Agric. 1988;46(2):147–173. 10.1002/jsfa.2740460204

[25] Hirono I, Fushimi K, Mori H, Miwa T, Haga M. Comparative study of carcinogenic activity in each part of bracken. J Natl Cancer Inst. 1973;50(5):1367–1371. Epub 1973/05/01. . 4712595 10.1093/jnci/50.5.1367

[26] Kirita T, Okabe S, Izumo T, Sugimura M. Risk factors for the postoperative local recurrence of tongue carcinoma. J Oral Maxillofac Surg. 1994;52(2):149–154. Epub 1994/02/01. . 8295049 10.1016/0278-2391(94)90398-0

[27] Shintani S, Matsuura H, Hasegawa Y, Nakayama B, Fujimoto Y. The relationship of shape of tumor invasion to depth of invasion and cervical lymph node metastasis in squamous cell carcinoma of the tongue. Oncology. 1997;54(6):463–467. Epub 1997/12/12. 10.1159/000227604 . 9394842

[28] Sato J, Yamazaki Y, Satoh A, Notani K, Kitagawa Y. Pain is associated with an endophytic cancer growth pattern in patients with oral squamous cell carcinoma before treatment. Odontology. 2010;98(1):60–64. Epub 2010/02/16. 10.1007/s10266-009-0107-6 . 20155509

[29] Eslami A, Miyaguchi K, Mogushi K, Watanabe H, Okada N, Shibuya H, et al PARVB overexpression increases cell migration capability and defines high risk for endophytic growth and metastasis in tongue squamous cell carcinoma. Br J Cancer. 2015;112(2):338–344. Epub 2014/11/26. 10.1038/bjc.2014.590 ; PubMed Central PMCID: PMCPMC4453450. 25422907 PMC4453450

[30] O-charoenrat P, Pillai G, Patel S, Fisher C, Archer D, Eccles S, et al Tumour thickness predicts cervical nodal metastases and survival in early oral tongue cancer. Oral Oncol. 2003;39(4):386–390. Epub 2003/04/05. . 12676259 10.1016/s1368-8375(02)00142-2

[31] Gonzalez-Moles MA, Esteban F, Rodriguez-Archilla A, Ruiz-Avila I, Gonzalez-Moles S. Importance of tumour thickness measurement in prognosis of tongue cancer. Oral Oncol. 2002;38(4):394–397. Epub 2002/06/22. . 12076706 10.1016/s1368-8375(01)00081-1

[32] Yuen APW, Lam KY, Lam LK, Ho CM, Wong A, Chow TL, et al Prognostic factors of clinically stage I and II oral tongue carcinoma—A comparative study of stage, thickness, shape, growth pattern, invasive front malignancy grading, Martinez-Gimeno score, and pathologic features. Head Neck-J Sci Spec. 2002;24(6):513–520. 10.1002/hed.10094. WOS:000176118400001. 12112547

[33] Masuda EK. Patogênese dos carcinomas de células escamosas alimentares associados ao consumo de *Pteridium aquilinum* em bovinos. PhD Thesis, Universidade Federal de Santa Maria, Brazil. 2010. Available from: http://w3.ufsm.br/ppgmv/images/MASUDA%20Eduardo%20Kenji%20II.pdf

[34] Duncan JR, Corbeil LB, Davies DH, Schultz RD, Whitlock RH. Persistent papillomatosis associated with immunodeficiency. Cornell Vet. 1975;65(2):205–211. Epub 1975/04/01. . 165037

[35] Munday JS. Bovine and human papillomaviruses: a comparative review. Vet Pathol. 2014;51(6):1063–1075. Epub 2014/07/02. 10.1177/0300985814537837 . 24981715

