# Supplementary material for: Bovine upper alimentary squamous cell carcinoma associated with bracken fern poisoning: Clinical-pathological aspects and etiopathogenesis of 100 cases
Source: PLoS One. 2018 Sep 26;13(9):e0204656. doi: 10.1371/journal.pone.0204656 (PMC6157896; doi:10.1371/journal.pone.0204656)
Supplement: S1 File — (PDF) [file pone.0204656.s001.pdf]

Supporting information

S1 File. In the absence of a commercially available specific primary antibody for BPV-4, a monoclonal antibody cocktail against human papillomavirus was used by immunohistochemistry and nuclear immunoreactivity for papillomavirus was not observed in any (n=93 papillomas of 38 cattle) of the UDT papillomas (Figure in S1 Fig. A and B).
