# Supplementary figures and images for: Bovine upper alimentary squamous cell carcinoma associated with bracken fern poisoning: Clinical-pathological aspects and etiopathogenesis of 100 cases
Source: PLoS One. 2018 Sep 26;13(9):e0204656. doi: 10.1371/journal.pone.0204656 (PMC6157896; doi:10.1371/journal.pone.0204656)

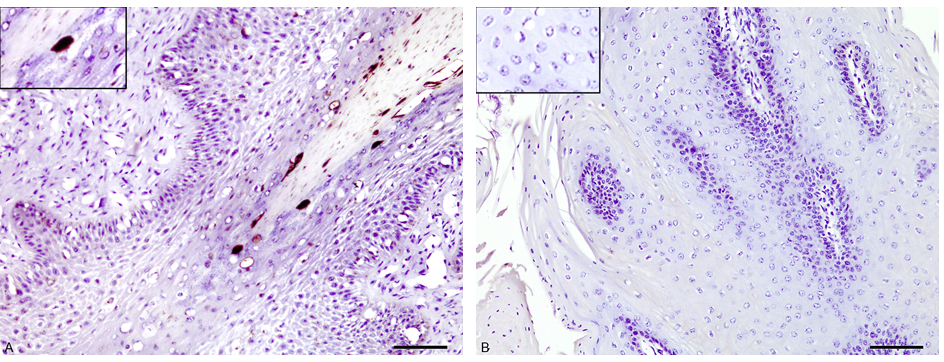

Supplement: S1 Fig — Nuclear immunoreactivity within the superficial keratinocytes in a bovine cutaneous papilloma used as positive control. Inset shows an immunolabelled keratinocyte (A). Absence of immunoreactivity in a bovine upper digestive tract papilloma. Inset shows absence of immunostaining in epithelial cells (B). (TIF) [file pone.0204656.s002.tif]
